# On the Introduction of the Vaccine Inoculation at Paris

**Published:** 1800-09

**Authors:** 


### *On the Introduction of the Vaccine Inoculation at Paris.*

Paris, le 14 Germinal (5 Avril, 1800.)

A Monſieur PEARSON, Médecin de l'Hopital de l'Inoculation Vaccine, à Londres.

LES Journaux nous ont fait connoître, Monſieur, les Eſſais faits en Angleterre, relativement à l'Inoculation de la Vaccine et dont les ſuccès ſont dus, *en grande partie*, *à votre* zèle eclairé. Quelques amis de l'humanité et de la ſcience, que vous cultivez avec tant de diſtinction, ont formè le projet d'introduire en France, une pratique qui paroit promettre de ſi grands avantages. Ils ont propoſé, a cet effet, une ſouſcription qui eſt ſur le point d'être remplie. *Vous en trouverez ci joint le proſpectus.* Mais pour mettre à éxécution les vuês qui y ſont expoſées, nous avons beſoin de votre intervention, et c'eſt avec confiance que nous la ſollicitons.

Quoique l'on ait commencé ici quelques eſſais ſur la Vaccine, dont l'ecole de médecine s'eſt occupèe, plus particulièrement, on n'a pû cependant parvenir encore a aucun réſultat poſitif, la difficulté de ſe procurer de la matière *aſſez récente*, en a ſans doute été la principale cauſe. Nos artiſtes ſemblent ne point connoître cette affection ſur les vaches. Dans une ſeule circonſtance, on a cru reconnaitre la maladie ſur des animaux de cette eſpéce, et on a ſaiſi cette occaſion de recueillir du virus, qui a été employé concurrement *avec de la matière reçue de Geneve et d'Angleterre*, *mais ſans aucun ſucces encore bien reconnu.* Nous nous trouvons donc prives du premier moyen néceſſaire pour commencer nos eſſais, et *c'eſt a vous que nous nous addreſſons pour nous le procurer.*

Nous deſirons, ſi toutes fois vous jugez ces précautions utiles, recevoir des croutes ou puſtules recueillies ſur les vaches, et ſur les ſujets inoculès, et des fils ou de petites eponges imprégnés de la matière, priſe ſur les uns et ſur les autres, de ces mêmes croutes ou puſtules fraiches et de l'ecoulement qui s'etablit à la plaie formée par l'affection locale de la partie inoculée: les differentes eſpeces de matières ou ſubſtances devroient être diſtinguées par une indication particulière. Si votre methode de conſerver les fils dans le gaz azote ou le gaz hydrogéne, vous paroit de quelqu' avantage, nous attendons *de votre zéle*, que vous voudrez bien l'employer. Nous prenons, avec notre miniſtre des relations extérieures, les meſures convenables, *pour que les moyens d'envoi les plus ſurs*, *les plus commodes*, *et ſur tout les plus prompts ſoient à votre diſpoſition.*

*ſition.* La ſociété ne mettra pas moins d'empreſſement, Monſieur, a faire connoître les ſervices que aurez bien voulu lui rendre et à vous informer du réſultat de ſes recherches, *dont le mérite vous ſera du en grande partie*, ſi à l'envoi de la matière que nous vous demandons, vous avez la bonté d'ajouter *quelques inſtructions particulieres*, dont nous nous feliciterons de pouvoir profiter.

*Salut*, *Eſtime* et *Devouement*, les Membres compoſant le Comité Médical de la Société formée à Paris, pour l'Inoculation de la Vaccine.

THOURET, Directeur de l'Ecole de Medecine.

PINEL, Prof. de l'Ecole de Medecine.

ROUSELLE CHAMSERU, Med. de l'Armée.

PARFAIT, Chir. Inoc.

TESSIER, de l'Inſtitut.

DE LA PORT, Med. des Hopit. Milit.

HUZARD, de l'Inſtitut.

CABANNEZ, de l'Inſtitut. et Prof. de l'Ecole de Med.

The above letter, as appears, was addreſſed to Dr. Pearſon, who, however, in place of returning an anſwer in his own name only, preſented it to the *Vaccine Inſtitution*, where it was determined that the commiſſion ſhould be executed by the *Medical Eſtabliſhment.* Accordingly a packet, containing vaccine matter on *glaſs*, on *lancets*, and on *thread*, incloſed in a bottle filled with hydrogen gas, and a letter of inſtructions, ſigned by all the members of the Medical eſtabliſhment, were ſent to Paris, on the 12th of May laſt, with the permiſſion of Lord Grenville, through the French Commiſſioner, Mr. Otto.

How well the vaccine matter, thus forwarded, anſwered the ſanguine expectations of the Pariſian Phyſicians, notwithſtanding the journey and delay, is ſufficiently proved by the following Extract from the Gazette Nationale; and other French papers furniſh ſimilar reports.

*Gazette Nationale*, 23 *Prarial*, *An.* 8, *de la Republique Françoise*, *une et indiviſible.*

Sur l'Inoculation de la Vaccine.

Le 13 Prarial l'Inoculation de la Vaccine à eu lieu ſur *trente Enfans*, avec la matière envoyée d'Angleterre et d'apres les procédes recommandés. Des ſignes d'infection ſe ſont maniſeſtés *sur neuf d'entre eux* aux époques et avec les caractères annonces par le Docteur Pearſon, et d'autres membres du Comité inſtitué à Londres pour l'Inoculation de la Vaccine. Il eſt a remarquer que les Medecins Anglois avoient exprimé, dans leur lettre, " Que l'on devoit ſe croire heureux, vu le

" laps

« laps de tems entre la collection de la matière et de fon ap-
« plication, fi, fur vingt individus inoculés, un feul prenoit la
« maladie.” Le 19, le 20, et le 21, *dix-huit enfans* fur lesquels la premiere Inoculation n'avoit point eu d'effet, ont été inoculés de nouveau avec la matière recueillie fur ceux chez qui la premiere inoculation avoit pris. Le Comité continue fes effais et inftruira le public des refultats ulterieurs.

Pour le Comité Médical,
THOURET.

---

VACCINE INOCULATION, from the Official Report at PARIS.

*Inoculation Houfe at Vaugirard, July* 27, 1800.

We have now got Dr. WOODVILLE, the Phyfician to the Inoculation Hofpital at London. This learned man, to whom we are indebted for an Effay on the Cow-pox, tranflated into French by Dr. Albert, animated by a generous zeal, and deferving in every refpect our gratitude and praife, is come to affift our experience in Vaccine Inoculation.

His practice has conftant and invariable fuccefs, as more than 6000 children inoculated by the cow-pox matter, and preferved from the Small-pox, promife us effects equally fatisfactory.

Does not this prefervative from the ufual difeafe feem, by its beneficent quality, to be a kind of prodigy, when we confider that the labour it gives rife to is nothing more than the puncture which one makes for the purpofe of inoculating, and is exempt from the flighteft accident.

COLON, M. D. Member of the Vaccine Medical Committee.

---

The Vaccine Inftitution, which has already been fo ufeful in England, and communicated with various parts of Europe, to introduce the Cow-pock Inoculation, has fent the following notice.

*Vaccine Inoculation Inftitution, Warwick Street, Golden Square.*
*July* 28, 1800.

From the increafe of patients, and the numerous applications for Vaccine matter, the Subfcribers are hereby informed, that it has been refolved to inoculate every Friday as well as Tuefday, under the direction of the Medical Eftablifhment, which at prefent is as follows:

G. PEARSON, M. D. F R. S. L. NIHELL, M. D. and T. NELSON, M. D. Phyficians.

THO. KEATE, Efq. F. R. S. and JOHN RUSH, Efq. Confulting Surgeons.

R. KEATE, Efq. J. GUNNING, Efq. and J. C. CARPUE, Efq. Surgeons.

A. BRANDE, Efq. F. RIVERS, Efq. and Mr. E. BRANDE, Vifiting Apothecaries.

Mr. J. LEWIS, Refident Apothecary, to whom letters (poft paid) may be addreffed.

The Public are defired to notice, that the Medical Eftablifhment do not authenticate Vaccine matter unlefs it be delivered under the appropriate feal of the Inftitution. By order,

W. SANCHO, Secretary.

